# Protocol for the Bottom-Up Proteomic Analysis of Mouse Spleen

**DOI:** 10.1016/j.xpro.2020.100196

**Published:** 2020-12-03

**Authors:** Paul Dowling, Stephen Gargan, Margit Zweyer, Michael Henry, Paula Meleady, Dieter Swandulla, Kay Ohlendieck

**Affiliations:** 1 Department of Biology, Maynooth University, National University of Ireland, Maynooth W23F2H6, Co. Kildare, Ireland; 2 Kathleen Lonsdale Institute for Human Health Research, Maynooth University, Maynooth W23F2H6, Co. Kildare, Ireland; 3 Department of Neonatology and Paediatric Intensive Care, Children’s Hospital, University of Bonn, D53113 Bonn, Germany; 4 National Institute for Cellular Biotechnology, Dublin City University, Dublin 9, Ireland; 5 Institute of Physiology II, University of Bonn, D53115 Bonn, Germany

**Keywords:** High Throughput Screening, Model Organisms, Protein Biochemistry, Proteomics, Mass Spectrometry

## Abstract

This protocol describes the comparative proteomic profiling of the spleen of wild type versus *mdx-4cv* mouse, a model of dystrophinopathy. We detail sample preparation for bottom-up proteomic mass spectrometry experiments, including homogenization of tissue, protein concentration measurements, protein digestion, and removal of interfering chemicals. We then describe the steps for mass spectrometric analysis and bioinformatic evaluation.

For complete details on the use and execution of this protocol, please refer to Dowling et al. (2020).

## Highlights

• Mass spectrometry-based proteomic analyses of spleen tissue samples
• Comparative profiling of the spleen from the *mdx-4cv* model of dystrophinopathy
• Protein extraction and protein digestion for peptide mass spectrometric analysis
• Bioinformatic analysis of large proteomic datasets for comparative studies

## Graphical Abstract

**Figure:**
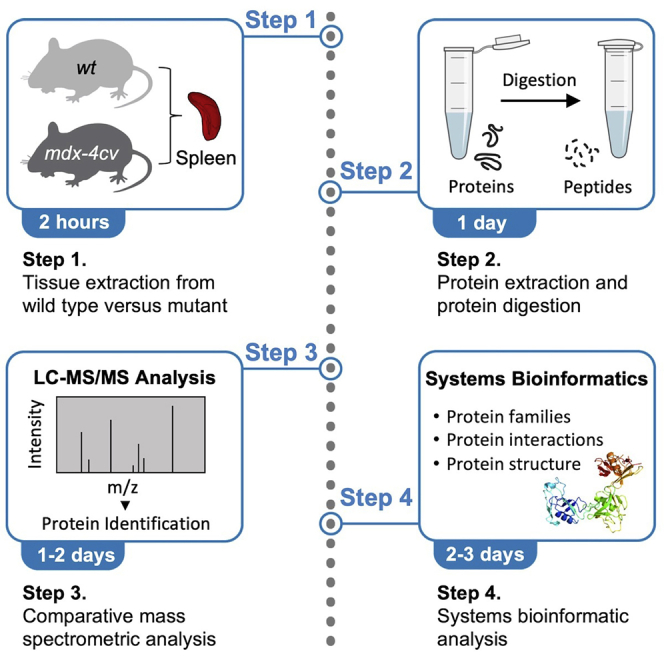

## Before You Begin

**Figure 1:**
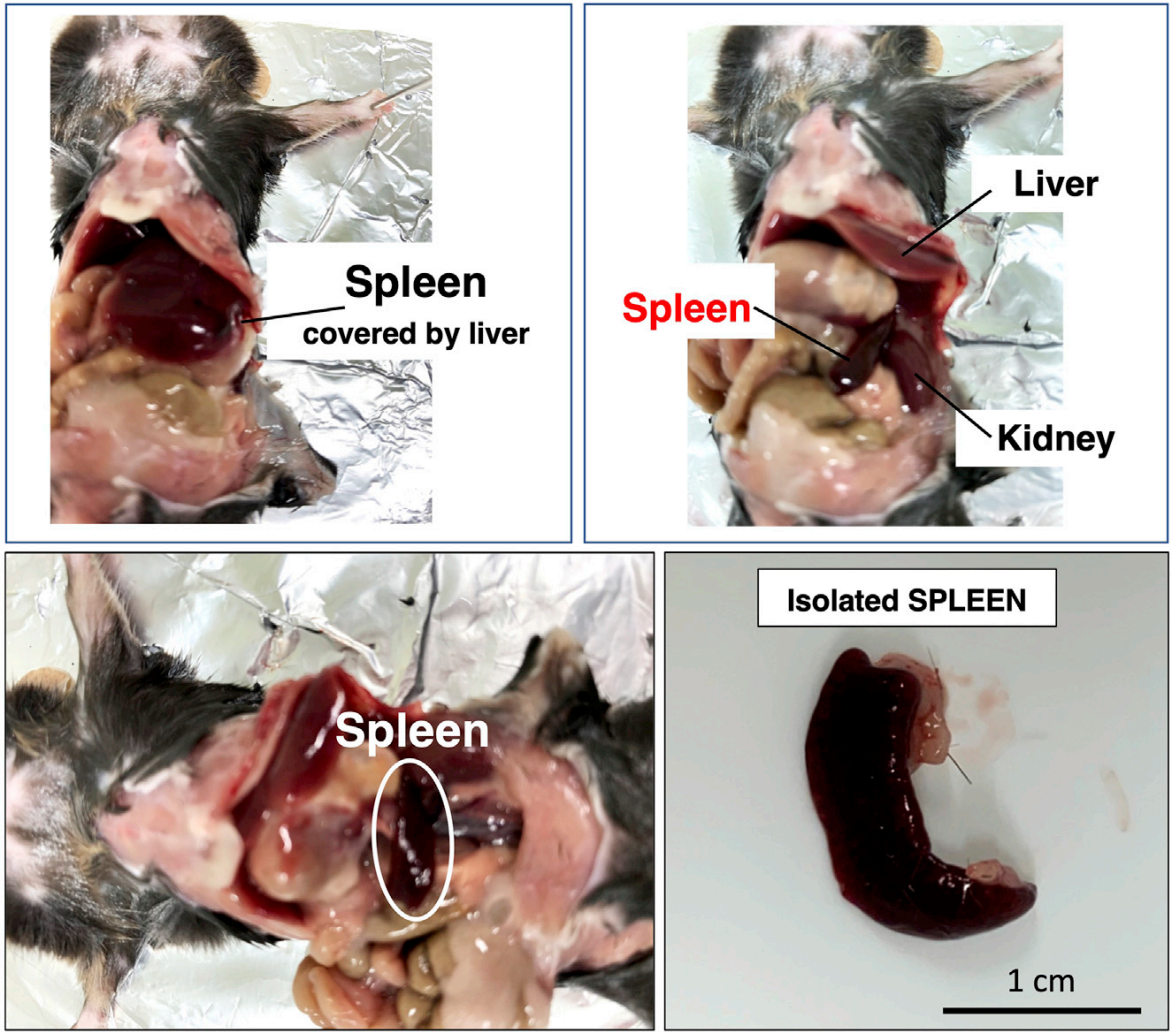
Overview of Dissection of Mouse Spleen

Prior to designing a specific proteomic workflow, it is crucial to initially investigate which biochemical screening approaches and protein identification methodologies are the most suitable way for the efficient analysis of the particular type of cell, tissue, or organ under investigation (Kang et al., 2020). In analytical protein biochemistry, the main high-throughput approaches can be divided into top-down proteomics versus bottom-up proteomics (Dupree et al., 2020). Besides antibody-based screening methods, the most frequently employed detection method for protein identification is mass spectrometry. Top-down proteomics focuses on the mass spectrometric analysis of intact proteoforms and is therefore the method of choice for investigating purified protein species and their post-translational modifications (Dowling et al., 2019). In contrast, bottom-up proteomics is a peptide-centric technique and highly useful for large-scale studies of protein abundance where peptides are produced and then analyzed by liquid chromatography-mass spectrometry (Manes and Nita-Lazar, 2018). Current techniques are highly efficient and allow for the rapid analysis of many sample types. This protocol represents a typical bottom-up proteomic workflow and was used in a recent publication (Dowling et al., 2020) to identify proteome-wide changes in the spleen of the dystrophic *mdx-4cv* mouse model of Duchenne muscular dystrophy. In Figure 1, the dissection of mouse spleen is shown. Ideally freshly dissected organs should be used for the initial preparatory step of comparative proteomic studies.

### Suitability of Animal Models and Tissue Specimens

Animals should be housed in a clean environment in a certified bioresource facility and kept under standard conditions (living space, light-dark cycles, nutrient availability). All procedures should adhere to local legislation on the use of animals in experimental research. For the comparative proteomic analysis of the spleen, freshly dissected organs from age- and gender-matched wild type C57/BL6 mice and dystrophic *mdx-4cv* mice should be quick-frozen in liquid nitrogen. For studying the effects of muscular dystrophy, *mdx-4cv* mice ranging in age from 1 to 12 months are suitable. Frozen tissue specimens can be transported on dry ice and be stored at −80°C for 12 months prior to biochemical analyses. In order to use the most suitable animal model and tissue specimens for studying specific pathophysiological aspects of a human disease by mass spectrometry-based proteomics, it is crucial to choose an appropriate genotype, phenotype, and tissue type for sample preparations. In the case of Duchenne muscular dystrophy, the most frequently inherited neuromuscular disorder of early childhood (Waldrop and Flanigan, 2019), the *mdx-4cv* mouse presents an excellent animal model that was generated by chemical mutagenesis (Chapman et al., 1989). This dystrophic mouse model of dystrophinopathy exhibits characteristic pathological hallmarks of skeletal muscle wasting throughout its lifetime, which includes myofiber degeneration, fat substitution, reactive myofibrosis, and sterile inflammation (Murphy et al., 2019). Progressive dysfunction has also been observed in a variety of other organs, such as the heart, brain, kidney, and liver (Murphy et al., 2018). The protocol described here using bottom-up proteomics to study detailed changes in a specific organ, i.e., the mass spectrometric analysis of the spleen from the *mdx-4cv* mouse (Dowling et al., 2020), has elucidated the importance of organ crosstalk in X-linked muscular dystrophy. This pathophysiological complexity of body-wide alterations makes the *mdx-4cv* mutant an appropriate mouse model to investigate proteome-wide changes in dystrophinopathy.

### Choice of Mass Spectrometric Approach

Based on extraordinary advancements in biochemical separation methods and drastic increases in the detection sensitivity of mass spectrometers, proteomics has developed into an indispensable tool for systems biological approaches (Uzozie and Aebersold, 2018). The main mass spectrometric research strategies in modern protein biochemistry can be divided into targeted proteomics versus discovery proteomics. Targeted proteomics is mostly concerned with the detection and absolute quantification of selected target peptides and proteins. In contrast, discovery proteomics focuses on large-scale protein screening and the analysis of protein dynamics in complex cellular systems. In this protocol, discovery proteomics was chosen to analyze differences in the protein expression profile of the *mdx-4cv* spleen (Dowling et al., 2020). For comparative analyses, proteomics, in combination with bioinformatics, can be used to swiftly determine spatial and temporal alterations in protein expression patterns. This is especially helpful for studying disease mechanisms and the identification of novel biomarker candidates. High-throughput mass spectrometric surveys of pathological tissue specimens can supply large datasets of proteome-wide changes due to pathophysiological abnormalities. It is therefore critical to choose the most suitable mass spectrometric method for the optimum biochemical analysis of the protein samples under investigation (Aebersold and Mann, 2016). Mass spectrometry-based proteomics that uses labeled or label-free proteins can be employed to determine the relative differences in protein abundances when comparing biological samples under different conditions (Kang et al., 2020). Routinely used labeling techniques include isotope-coded affinity tags (ICAT), tandem mass tags (TMT), isobaric tags for relative and absolute quantitation (iTRAQ) and stable isotope labeling by amino acids in cell culture (SILAC), especially useful in the analysis of cell line models (Craft et al., 2013). Limitations of using labeling approaches include cost per experiment, challenging sample preparation and, in some instances, difficulties with the efficiency of protein labeling. However, for certain proteomic analyses, especially the analysis of post-translational modifications, labeling approaches are an excellent choice. In label-free quantitation, using peak area and/or spectral counting, the variability that chemical labeling/tagging may introduce is eliminated, the protocol is more cost-effective and sample preparation in many cases, is more simplified. All considerations associated with both labeled or label-free approaches need to be taken into account before a specific strategy is decided on. The field of mass spectrometry is forever pushing the boundaries with advanced technologies such as targeted labeling including MRM (Percy et al., 2017) and PRM (Wilson et al, 2019) approaches, targeted label-free SWATH (Muench et al., 2020) techniques and improved data-acquisition. Suitable instrument and experimental designs are available with discovery free-label BoxCar methodology (Meier et al., 2018). For this protocol, peptide separation and identification were achieved by combining reverse-phased capillary high-pressure liquid chromatography and the analytical capability of an Orbitrap Fusion Tribrid mass spectrometer (Thermo Fisher Scientific, Waltham, MA, USA).

### Buffer Preparation

**Timing: 3–5 h**

1. All essential buffers and solutions should be freshly made prior to sample preparation and used the same day to avoid any potential degradation of key chemicals. Make sure that there is enough of all solutions that are required for tissue homogenization, assessment of protein concentration, protein digestion, removal of interfering chemicals, liquid chromatography, and mass spectrometry.
2. All solutions should be prepared with analytical grade chemicals that are suitable for LC-MS analysis and ultrapure water.
3. Buffers for the initial preparation of tissue extracts should be supplemented with a commercially available protease inhibitor cocktail to avoid the degradation of sensitive proteins.
4. During all analytical steps, protective gloves, laboratory coat, and face mask should be worn, especially during preparation of sodium dodecyl sulfate and iodoacetamide containing buffers.

## Key Resources Table

**Table:** 

REAGENT or RESOURCE	SOURCE	IDENTIFIER
**Chemicals, Peptides, and Recombinant Proteins**		

Ammonium bicarbonate	Sigma	A6141
Sodium dodecyl sulfate	Sigma	L3771
Tris base	Sigma	T1503
Dithiothreitol	Thermo Fisher Scientific	BP172-5
Iodoacetamide	Acros Organics	122270050
Trypsin protease, MS grade	Thermo Fisher Scientific	90305
Trifluoroacetic acid	Sigma	T6508
Acetonitrile	Sigma	34851
Formic acid	Sigma	5330020050
Urea	Sigma	U0631
LC-MS grade water	Sigma	39253
Bovine serum albumin	Thermo Fisher Scientific	23208

**Critical Commercial Assays**		

Pierce 660 nm Protein Assay Reagent	Thermo Fisher Scientific	1861426
Ionic Detergent Compatibility Reagent for Pierce 660 nm Protein Assay Reagent	Thermo Fisher Scientific	22663
Halt Protease Inhibitor Cocktail (100×)	Thermo Fisher Scientific	78429

**Deposited Data**		

Open Science Foundation	OSF	osf.io/f85ve

**Experimental Models: Organisms/Strains**		

C57/BL6 mice	Jackson Laboratory	000664
B6Ros.Cg-Dmd^mdx-4Cv^/J mice	Jackson Laboratory	002378

**Software and Algorithms**		

Proteome Discoverer 2.2 using Sequest HT	Thermo Fisher Scientific	OPTON-30945
Progenesis QI for Proteomics	Waters Chromatography Ireland Ltd.	n/a

**Other**		

Kinematica Polytron PT1200E handheld homogenizer	Thermo Fisher Scientific	08-451-164
Filter unit Vivacon 500	Sartorius	VN0H22
Pierce C18 spin columns	Thermo Fisher Scientific	89870
ThermoMixer	Eppendorf	5382000031
Benchtop centrifuge	Eppendorf	5427R
Sonicator	Bandelin	UW2200
Vortex	Sigma	Z258423
Incubator	Memmert	INB200
Vacuum evaporator	Genevac	DNA-12060-C00
Microplate reader	Thermo Fisher Scientific	VL0000D0
Reverse-phased capillary high-pressure liquid chromatography system	Thermo Fisher Scientific	UltiMate 3000 HPLC
Orbitrap Fusion Tribrid mass spectrometer	Thermo Fisher Scientific	IQLAAEGAAPFADBMBCX
Heated electrospray ionization (H-ESI) ion source	Thermo Fisher Scientific	H-ESI probe

## Materials and Equipment

**Table:** Tris buffer

Reagent	Final concentration	Volume
Tris buffer	0.1 M Tris, pH 7.8	1,000 mL

Dissolve 12.11 g of Tris in distilled water and make up to a total volume of 1,000 mL with distilled water. Adjust pH to 7.8 with HCl.

**Table:** Ammonium bicarbonate buffer

Reagent	Final concentration	Volume
Ammonium bicarbonate buffer	50 mM ammonium bicarbonate in 0.1 M Tris buffer, pH 7.8	500 mL

Dissolve 1.95 g of ammonium bicarbonate in Tris buffer and make up to a total volume of 500 mL with Tris buffer.

**Table:** Homogenization buffer

Reagent	Final concentration	Volume
Homogenization buffer	1% (w/v) sodium dodecyl sulfate, 0.1 M dithiothreitol in 50 mM ammonium bicarbonate buffer	100 mL

Dissolve 1 g of sodium dodecyl sulfate and 1.54 g of dithiothreitol in 50 mM ammonium bicarbonate buffer and make up to a total volume of 100 mL with 50 mM ammonium bicarbonate buffer, pH 7.8.

**CRITICAL:** Sodium dodecyl sulfate is an eye, skin, and respiratory irritant. To prevent exposure, make sure to wear suitable protective gloves, as well as protective clothing, eye protection, a mask, and proper face protection during the handling of sodium dodecyl sulfate in its powder form.

**Table:** Urea buffer

Reagent	Final concentration	Volume
Urea buffer	8 M urea in 50 mM ammonium bicarbonate buffer	250 mL

Dissolve 120.12 g of urea in 50 mM ammonium bicarbonate buffer and make up to a total volume of

250 mL with 50 mM ammonium bicarbonate buffer.

**Table:** Iodoacetamide solution

Reagent	Final concentration	Volume
Iodoacetamide solution	50 mM iodoacetamide in urea buffer	5 mL

Dissolve 46 mg of iodoacetamide in urea buffer and make up to a total volume of

5 mL with 8 M urea buffer.

**CRITICAL:** Iodoacetamide is considered hazardous by the Hazard Communication Standard (29 CFR 1910.1200). According to chemical safety data information, iodoacetamide is classified as acute toxic, irritant, and a health hazard. To prevent exposure, make sure to wear suitable protective gloves, as well as protective clothing, eye protection, a mask, and proper face protection.

**Table:** Digestion buffer

Reagent	Final concentration	Volume
Digestion buffer	50 mM ammonium bicarbonate with trypsin (50:1 protein:trypsin ratio)	5 mL

Dissolve 20 ng of trypsin (per 1,000 ng of protein) in ammonium bicarbonate buffer and make up to a total volume of 5 mL with 50 mM ammonium bicarbonate buffer.

**Table:** Sample buffer

Reagent	Final concentration	Volume
Sample buffer	2% (v/v) trifluoroacetic acid, 20% (v/v) acetonitrile	20 mL

Gently mix 0.4 mL of trifluoroacetic acid with 4 mL of acetonitrile and make up to a total volume of 20 mL with LC-MS grade water.

**Table:** Activation solution

Reagent	Final concentration	Volume
Activation solution	50% (v/v) acetonitrile	20 mL

Gently mix 10 mL of acetonitrile with LC-MS grade water and make up to a total volume of 20 mL with LC-MS grade water.

**Table:** Equilibration/wash solution

Reagent	Final concentration	Volume
Equilibration/wash solution	0.5% (v/v) trifluoroacetic acid in 5% (v/v) acetonitrile	50 mL

Gently mix 0.25 mL of trifluoroacetic acid with 2.5 mL of acetonitrile and make up to a total volume of 50 mL with LC-MS grade water.

**Table:** Elution buffer

Reagent	Final concentration	Volume
Elution buffer	80% (v/v) acetonitrile	5 mL

Gently mix 4 mL of acetonitrile with LC-MS grade water and make up to a total volume of 5 mL with LC-MS grade water.

**Table:** Resuspension buffer

Reagent	Final concentration	Volume
Resuspension buffer	2% (v/v) acetonitrile, 0.1% (v/v) trifluoroacetic acid	5 mL

Gently mix 0.1 mL of acetonitrile with 5 μL of trifluoroacetic acid and make up to a total volume of 5 mL with LC-MS grade water.

**Table:** Trapping buffer

Reagent	Final concentration	Volume
Trapping buffer	2% (v/v) acetonitrile, 0.1% (v/v) trifluoroacetic acid	1,000 mL

Gently mix 20 mL of acetonitrile with 1 mL of trifluoroacetic acid and make up to a total volume of 1,000 mL with LC-MS grade water.

**Table:** LC – Solvent A

Reagent	Final concentration	Volume
LC - Solvent A	0.1% (v/v) formic acid in LC-MS grade water	1,000 mL

Gently mix 1 mL formic acid with LC-MS grade water and make up to a total volume of 1,000 mL with LC-MS grade water.

**Table:** LC – Solvent B

Reagent	Final concentration	Volume
LC - Solvent B	80% (v/v) acetonitrile, 0.08% (v/v) formic acid in LC-MS grade water	1,000 mL

Gently mix 800 mL of acetonitrile with 0.8 mL of formic acid and make up to a total volume of 1,000 mL with LC-MS grade water.

## Step-By-Step Method Details

### Tissue Homogenization

**Timing: 2–3 h**

Prior to protein extraction for mass spectrometric analysis, a crucial step of comparative proteomic studies is the reproducible homogenization of the biological material of interest. In the case of small tissue/organ specimens from mice such as the spleen, a handheld device is in our experience ideal for swift and efficient tissue homogenization in small sample volumes (Dowling et al., 2020). Various types of handheld devices are available. In this protocol, the Kinematica Polytron model PT1200E was used for the efficient homogenization of mouse spleen specimens. During the initial preparation of tissue extracts, buffers should be supplemented with a suitable protease inhibitor cocktail to avoid the degradation of potentially sensitive proteins. Treatment at 95°C is a suitable approach to counteract the masking effect that highly abundant proteins such as albumin have with respect to maximizing the number of proteins identified in biological samples (Chiangjong et al., 2019). High temperature precipitation of thermolabile proteins is a relatively straightforward step and compares favorably to optimize demanding protocols for high abundant protein removal such as immunodepletion. For mass spectrometric analyses, sufficient biological and technical repeats should be used for the statistical evaluation of protein hits. Ideally, n=6 biological repeats and n=2 technical repeats per specimen of interest are employed to account for potential biological and bioanalytical variations.

1. Homogenize mouse spleen tissue (25 mg wet weight) using a handheld homogenizer in 150 μL of homogenization buffer, supplemented with 1× protease inhibitor cocktail. The ratio of tissue to buffer should be 1:6 (mg/μL).
2. Heat the suspension at 95°C for 5 min.
3. Sonicate the suspension using 4 bursts of 5 s to shear the DNA to reduce the viscosity of the sample.
4. Centrifuge the suspension at 20,000 × *g* for 10 min to clarify the homogenate.
5. Determine protein concentration using the Pierce 660 nm protein assay kit.

### Protein Determination

**Timing: 1–2 h**

For comparative proteomic studies, it is crucial to accurately determine the protein concentration in individual samples. This can be conveniently carried out with a variety of commercially available protein assays. In this protocol, the Pierce 660 nm Protein Assay system (Antharavally et al., 2009) and pre-prepared protein standards were employed for the swift determination of protein concentration. Since the protocol described here uses detergents during the homogenization step, ionic detergent compatibility reagent has to be added to the protein assay reagent.

6. Add 10 μL of each protein standard, such as bovine serum albumin (25, 50, 125, 250, 500, 750, 1,000, 1,500 and 2,000 μg/mL), the unknown samples and the appropriate blank sample (homogenization buffer) into a microplate well in triplicate.
7. Add 150 μL of the protein assay reagent (supplemented with ionic detergent compatibility reagent) to each well.
8. Cover plate and mix on a plate shaker at medium speed (of approximately 600 rpm) for 1 min and incubate at 20°C for 5 min.
9. Use the blank wells to zero the plate reader. Measure the absorbance of the standards and unknown samples at a wavelength of λ = 660 nm.
10. Prepare a standard curve by plotting the average blank-corrected 660 nm measurement for each bovine serum albumin standard versus its concentration in μg/mL.
11. Use the standard curve to determine the protein concentration of each unknown sample, using a four-parameter (quadratic) curve fit.

### Protein Digestion

**Timing: 10–24 h**

**Figure 2:**
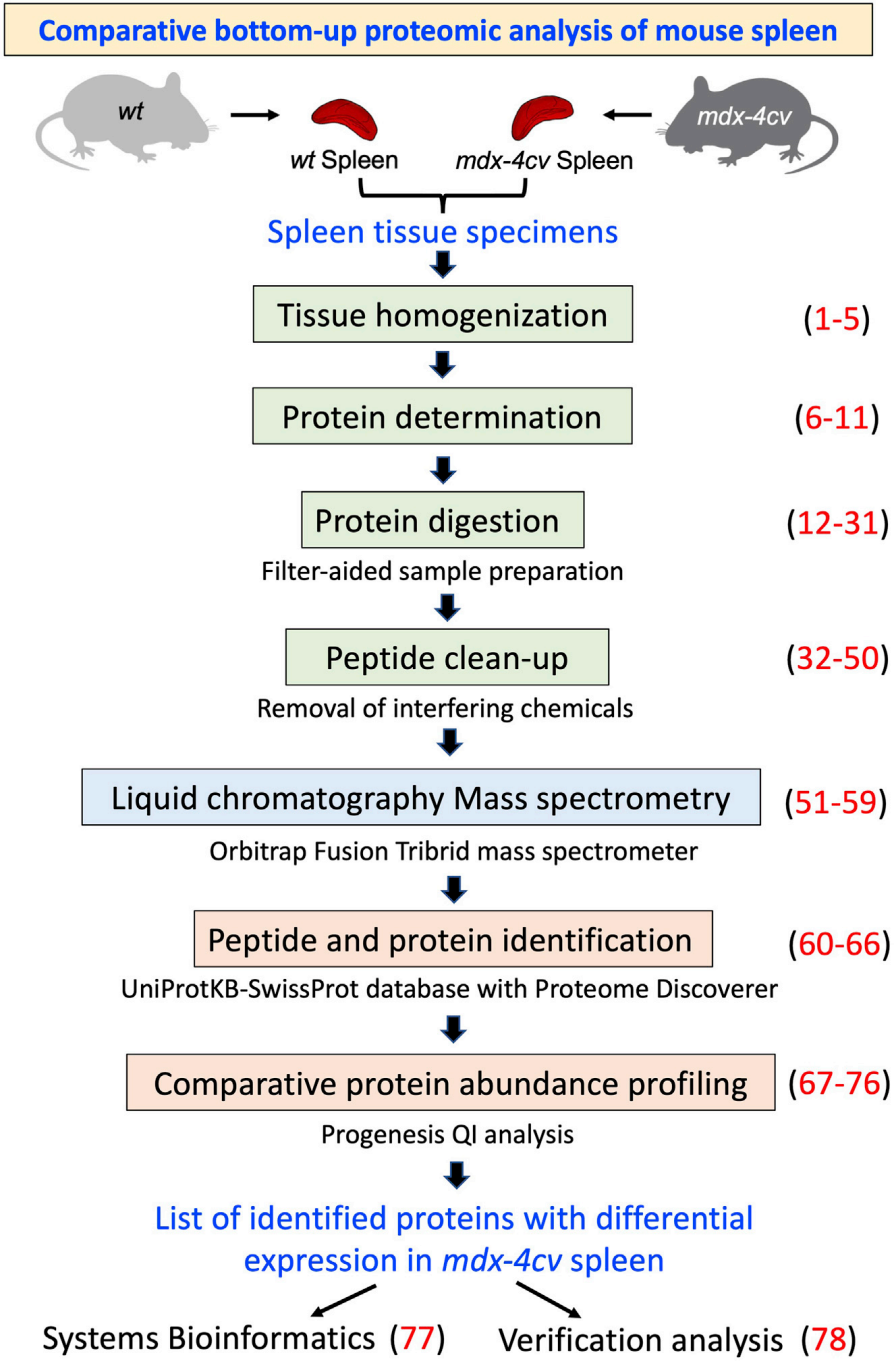
Workflow of the Comparative and Mass Spectrometry-Based Proteomic Profiling of the Spleen from Wild Type versus the Dystrophic *mdx-4cv* Mouse Model of Duchenne Muscular Dystrophy The corresponding experimental steps are listed in parentheses.

A key step of bottom-up proteomics is the controlled digestion of protein mixtures with a suitable protease. The most commonly used protease is trypsin and is also employed in this protocol. Alternative digestion strategies can be carried out with the enzyme Lys-C or combinations of Lys-C and trypsin, as well as a variety of other proteases or chemicals (Murphy and Ohlendieck, 2018). The workflow of tissue homogenization, protein determination, and protein digestion, as well as the subsequent peptide mass spectrometric analysis, protein identification, and bioinformatic analysis, is summarized in the flowchart of Figure 2. In this protocol, a filter-aided sample preparation approach, usually abbreviated as FASP, is used for processing the sodium dodecyl sulfate-solubilized spleen cells in a centrifugal filter unit (Wiśniewski, 2018). The disposable centrifugal ultrafiltration units can be conveniently used for the sequential removal of excess detergent, the controlled trypsination of proteins and the subsequent isolation of generated peptide populations. Prior to conducting filter-aided sample preparations, the appropriate cutoff size of the filter units (e.g., 10,000, 30,000 or 50,000 MWCO) should be evaluated for particular samples and types of analyses. For optimum chromatographical separation and subsequent mass spectrometric analysis, the choice of molecular weight cutoff should on the one hand prevent the retention of interfering chemicals and on the other hand not result in the loss of peptides/proteins. In our experience, the usage of smaller pore size cutoff filters (10 kDa) results in the retention of chemicals that can alter chromatography. Using a filter with a 30 kDa pore size reduces this interference greatly resulting in more reproducible data.

12. Combine 25 μg of sample homogenate (maximum volume of 30 μL) with 200 μL urea buffer in the Sartorius Vivacon 500 centrifugal filter unit.
13. Briefly vortex and centrifuge at 14,000 × *g* for 15 min.
14. Add 200 μL of urea buffer to the filter unit, briefly vortex and then centrifuge at 14,000 × *g* for 15 min. Discard the flow-through solution.
15. Add 100 μL of iodoacetamide solution. This solution is light sensitive. Therefore, the filter units should be covered in tinfoil.
16. Mix this solution at 600 rpm in a thermomixer for 1 min and incubate without mixing for 20 min in the dark.
17. Centrifuge at 14,000 × *g* for 15 min.
18. Add 100 μL urea buffer to the filter unit.
19. Briefly vortex and centrifuge at 14,000 × *g* for 15 min.
20. Repeat this step by adding 100 μL urea buffer, followed by vortexing and centrifugation at 14,000 × *g* for 15 min.
21. Add 100 μL of ammonium bicarbonate buffer.
22. Briefly vortex and centrifuge at 14,000 × *g* for 15 min.
23. Repeat this step by adding 100 μL ammonium bicarbonate buffer, followed by vortexing and centrifugation at 14,000 × *g* for 15 min.
24. Transfer the filter units to new collection tubes.
25. Add 40 μL of trypsin-containing digestion buffer and keep this solution on ice.
26. Mix the solution at 600 rpm in a thermomixer for 1 min.
27. Incubate the filter units in a wet chamber. This can be constructed with a pipette box containing water and soaked tissue sheets and tubes held in foam floater, as illustrated in Figure 3. Incubation should be carried out in a sterile incubator at 37°C for 4–18 h.

**Figure 3:**
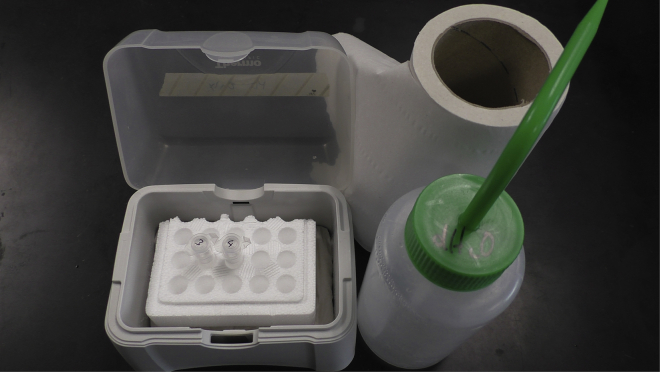
Image of a Pipette Box Containing Water and Soaked Tissue Sheets and Filter Unit Tubes Held in Foam Floater for Incubation in a Wet Chamber (Step 27)
28. Place filter units in new collection tubes.
29. Collect the peptide-containing filtrate by centrifugation at 14,000 × *g* for 10 min.
30. Add 40 μL of ammonium bicarbonate buffer and re-centrifuge the filter units at 14,000 × *g* for 10 min.
31. Add 60 μL of each sample to new Eppendorf tubes and add 15 μL of sample buffer (containing 2% (v/v) trifluoroacetic acid and 20% (v/v) acetonitrile), so that the peptide sample solution contains 0.5% trifluoroacetic acid in 5% acetonitrile.

### Peptide Clean Up to Remove Interfering Chemicals

**Timing: 1–3 h**

In order to remove interfering chemicals prior to mass spectrometric analysis, peptide-containing samples are washed and centrifuged prior to elution and liquid chromatographical separation.

32. Place C18 spin tubes into receiver tubes.
33. Add 200 μL of Activation Solution to rinse walls of the C18 spin tubes and to wet resin.
34. Centrifuge at 1,500 × *g* for 1 min and discard flow-through solution.
35. Repeat steps 33 and 34.
36. Add 200 μL Equilibration/Wash Solution.
37. Centrifuge at 1,500 × *g* for 1 min and discard flow-through solution.
38. Repeat steps 35 and 36.
39. Pipette sample on top of resin bed.
40. Place C18 spin tubes into receiver tubes.
41. Centrifuge at 1,500 × *g* for 1 min.
42. To ensure complete binding, recover flow-through and repeat steps 39–41.
43. Place C18 spin tubes into new receiver tubes.
44. Add 200 μL Equilibration/Wash Solution to C18 spin tube and centrifuge at 1,500 × *g* for 1 min and discard flow-through solution.
45. Repeat step 44.
46. Place C18 spin tubes in new receiver tubes.
47. Add 20 μL of Elution Buffer to top of the resin bed.
48. Centrifuge at 1,500 × *g* for 1 min.
49. Repeat steps 47 and 48 with same receiver tubes.
50. Gently dry sample in a vacuum evaporator and resuspend sample in 50 μL of resuspension buffer, so that the peptide sample solution contains 2% (v/v) acetonitrile and 0.1% (v/v) trifluoroacetic acid.

### Liquid Chromatography-Mass Spectrometry Using Orbitrap Fusion Tribrid

**Timing: 12–72 h (depending on the number of samples to be analyzed by LC-MS)**

A key bioanalytical step of bottom-up proteomics is the peptide-centric identification of protein species. Peptides generated by trypsin treatment are separated by reverse-phased liquid chromatography and then analyzed by mass spectrometry (Sethi et al., 2015). The amino acid sequence information of individual peptides is combined and then used for the systematic identification of individual proteoforms.

51. Using a liquid chromatography system (for example the Thermo Scientific UltiMate 3000 UHPLC), load 2 μL of protein digest onto the trapping column (PepMap100, C18, 300 μm × 5 mm - Thermo Fisher Scientific) at a flow rate of 25 μL/min with trapping buffer for 3 min before being resolved onto an analytical column (Acclaim PepMap 100, 75 μm × 50 cm, 3 μm bead diameter column).
52. Elution of peptides is carried out with the following binary gradient; LC Solvent A and LC Solvent B using 2%–32% Solvent B for 75 min, 32%–90% Solvent B for 5 min and holding at 90% for 5 min at a flow rate of 300 nL/min.
53. For peptide identification analysis, a data-dependent acquisition method is selected using a voltage of 2.0 kV and a capillary temperature of 320°C.
54. Data-dependent acquisition with full scans in the 380–1,500 m/z range is performed using an mass analyzer (for example the Thermo Scientific Orbitrap Fusion Tribrid) with a resolution of 120,000 (at m/z 200), a targeted automatic gain control (AGC) value of 4E+05 and a maximum injection time of 50 ms.
55. The number of selected precursor ions for fragmentation is determined by the top-speed acquisition algorithm.
56. Selected precursor ions are isolated in the Quadrupole with an isolation width of 1.6 Da.
57. Peptides with a charge state of 2+ to 7+ are analyzed and a dynamic exclusion is applied after 60 s.
58. Precursor ions are fragmented using higher energy collision-induced dissociation with a normalized collision energy of 28%. Resulting MS/MS ions are measured in the linear ion trap.
59. The typical MS/MS scan conditions are as follows: a targeted AGC value of 2E+04 and a maximum fill time of 35 ms.

### Peptide and Protein Identification

**Timing: 2–3 h**

In order to analyze mass spectrometric files, commercially available software programs can be used. It is crucial to define suitable search parameters for proper protein identification.

60. For data analysis of mouse proteins, mass spectrometric files (.raw) are searched against the UniProtKB-SwissProt *Mus musculus* database with Proteome Discoverer 2.2 using Sequest HT and Percolator.
61. Locate the proteome for your organism of interest by searching by name or by taxonomy ID (i.e., *Mus musculus* for Mouse) at https://www.uniprot.org/proteomes/.
62. Click on the Proteome ID link - UP000000589.
63. UniProt offers different databases to select from, including reviewed (UniProtKB/Swissprot), unreviewed (UniProtKB/TREMBL) or both (UniProtKB) options. In model organisms such as mice, which are researched extensively, reviewed (Swissprot) databases contain highly curated (i.e., carefully annotated) entries, a minimal level of redundancy and the ability to integrate with other databases efficiently.
64. Click on the Download button and choose: All protein entries, Fasta (Canonical and isoform), compressed.
65. The following search parameters are a useful guide for protein identification:
  a. Peptide mass tolerance set to 10 ppm.
  b. MS/MS mass tolerance set to 0.6 Da.
  c. Up to two missed peptide cleavages should be allowed.
  d. Carbamidomethylation of cysteine set as a fixed modification.
  e. Methionine oxidation set as a variable modification.
66. Importantly, only highly confident peptide identifications with a false discovery rate (FDR) ≤ 0.01, identified using a SEQUEST HT workflow coupled with Percolator validation in Proteome Discoverer 2.2, should be considered.

### Comparative Protein Abundance Profiling Using Progenesis QI Analysis

**Timing: 6–48 h**

The comparative proteomic analysis of different protein populations can be carried out by commercially available software programs. For example, protein extracts of tissue preparations from wild type versus mutant specimens can lead to the identification of hundreds of differentially expressed protein species. This protocol is based on the recent proteomic profiling of the spleen from normal versus dystrophic *mdx-4cv* mice (Dowling et al., 2020).

67. Using Progenesis QI (Waters) for comparative abundance profiling, mass spectrometric raw files are imported into the software and automatic run alignment can be used to combine and compare the result from different LC-MS runs.
68. Automatic peak picking and matching across all data files is carried out to create an aggregate dataset from the aligned runs. For a representative example, see Figure 4.

**Figure 4:**
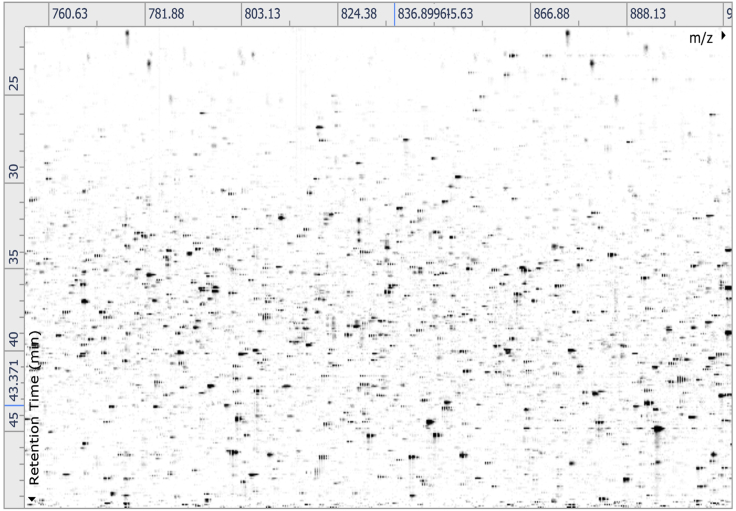
Aggregate Dataset from Aligned Runs Shown is a representative example of a reference run that other samples are aligned to (step 68).
69. This contains all peak information from all sample files used and allows the detection of a single map of peptides.
70. This map is then applied to each sample, giving 100% matching of peaks with no missing values.
71. The peptide ion abundance measurements are normalized allowing for the comparisons between the wild type and the mutant specimens in order to identify peptides of biological interest. For a representative example, see Figure 5.

**Figure 5:**
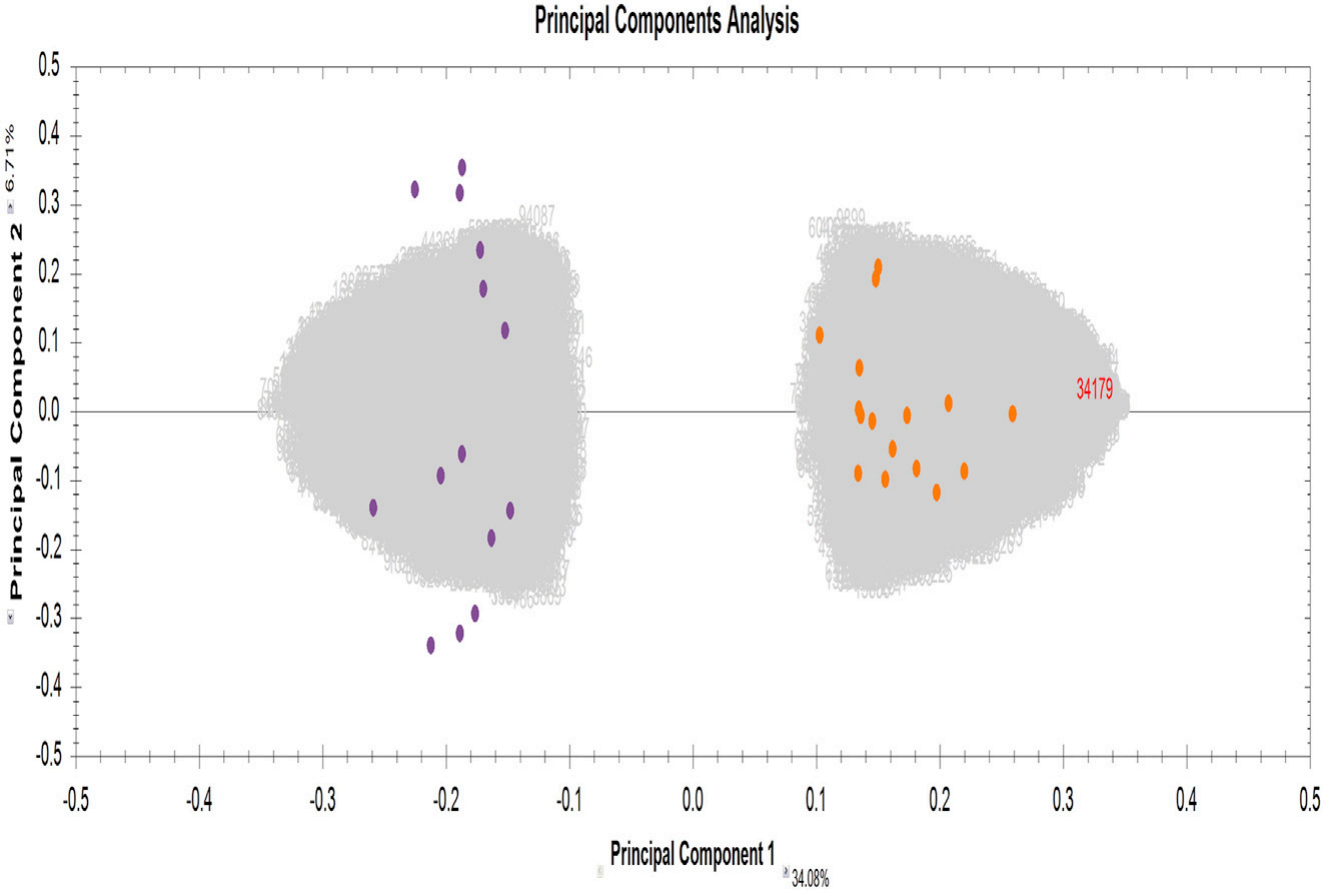
Comparisons between Wild Type and Mutant Specimens in Order to Identify Peptides of Biological Interest Shown is a representative example of clustering (*wt* samples marked in purple vs *mdx-4cv* samples marked in orange) based on significant peptides (step 71).
72. The peptide ions of interest are determined based on the significance measure of ANOVA with a p-value of 0.05.
73. MS/MS spectra from these peptides are exported and identified using the process above using Proteome Discoverer 2.2 software. For user guides and tutorial datasets of Progenesis QI for proteomics, see: http://www.nonlinear.com/progenesis/qi-for-proteomics/v4.0/user-guide/).
74. The result file is re-imported in Progenesis QI which allows a review of all the peptide ions used to quantify and identify individual protein species.
75. The proteins of interest are then determined based on the significance measure of ANOVA with a p-value of 0.05.
76. It is crucial to review quality control metrics before finalizing the list of significant proteins with a changed abundance. Critical metrics to be considered include:
  a. Sample preparation metrics – highlighting issues or problems with the preparation of your samples, for example problems due to missed peptide cleavages.
  b. Instrument metrics – highlighting whether your chromatography column and mass spectrometer are configured and performing correctly, for example issues with column deterioration.
  c. Experiment metrics – concerning the identified proteins and peptides in your experiment, for example the identification of statistical outliers in the sample set.

### Systems Bioinformatics

**Timing: days to weeks**

For further analyses of proteomic datasets and sequence information of individual proteoforms of biological or pathophysiological interest, a large variety of publicly accessable bioinformatics programs are available. Bioinformatics can be used to model protein structures, determine the distribution of distinct protein families within large proteomic datasets, evaluate the affiliation of individual proteins to biochemical pathways or protein assemblies, and establish potential protein-protein interaction patterns between identified proteoforms (Na and Paek, 2020). Below listed are commonly employed search programs for the systematic analysis of proteomic data.

77. Proteomic datasets and peptide sequences can be further analyzed by a variety of bioinformatic software programs, such as:
  a. UniProt (https://www.uniprot.org): Comprehensive database of protein sequence and functional information.
  b. BLAST (https://blast.ncbi.nlm.nih.gov): BLASTP is an algorithm and search program for comparing amino acid sequences of proteins.
  c. PANTHER (http://www.pantherdb.org): The PANTHER classification system is a large curated biological database of gene/protein families and their functionally related subfamilies that can be used to classify and identify the function of gene products.
  d. STRING (https://string-db.org): Program for the identification of protein-protein interaction networks and functional enrichment analysis.
  e. REACTOME (www.reactome.org): Database that provides a bioinformatics tools for the visualization and analysis of biological pathways.
  f. piNET (www.pinet-server.org): Server for peptide-centric post-translational modification mapping, differential expression analysis, gene (protein) enrichment analysis, and Library of Integrated Network-based Cellular Signatures (LINCS) connectivity mapping.
  g. DAVID (https://david.ncifcrf.gov): Database for annotation, visualization, and integrated discovery that provides a comprehensive set of functional annotation tools for studying the biological meaning of large lists of genes.
  h. KEGG (https://www.genome.jp/kegg/): Database resource for studying high-level functions and utilities of biological systems using large-scale molecular datasets.
  i. Phyre2 (http://www.sbg.bio.ic.ac.uk/∼phyre2): Automatic fold recognition server for predicting the structure of peptide and protein sequences.

### Verification Analysis

**Timing: weeks to months**

Mass spectrometry-based proteomics is an unbiased and technology-driven approach in exploratory bioresearch. Thus, the design of a comparative proteomic study is not necessarily based on a specific biological hypothesis, but is instead valuable in formulating novel research questions. Proteomic findings can therefore form the rationale for new bioanalytical avenues in a specific area of research. This frequently includes crucial and independent verification experiments to confirm proteomic findings (Dowling et al., 2020). Below listed are a few of the most commonly performed experiments to further investigate the biological or pathophysiological meaning of findings from proteomic surveys.

78. To confirm and interpret proteomic hits or data from comparative mass spectrometric studies, a large variety of standard biochemical and cell biological techniques are used, including:
  a. Immunoblotting
  b. Enzyme assays
  c. Protein binding assays
  d. Immunofluorescence microscopy
  e. Physiological assays

## Expected Outcomes

**Figure 6:**
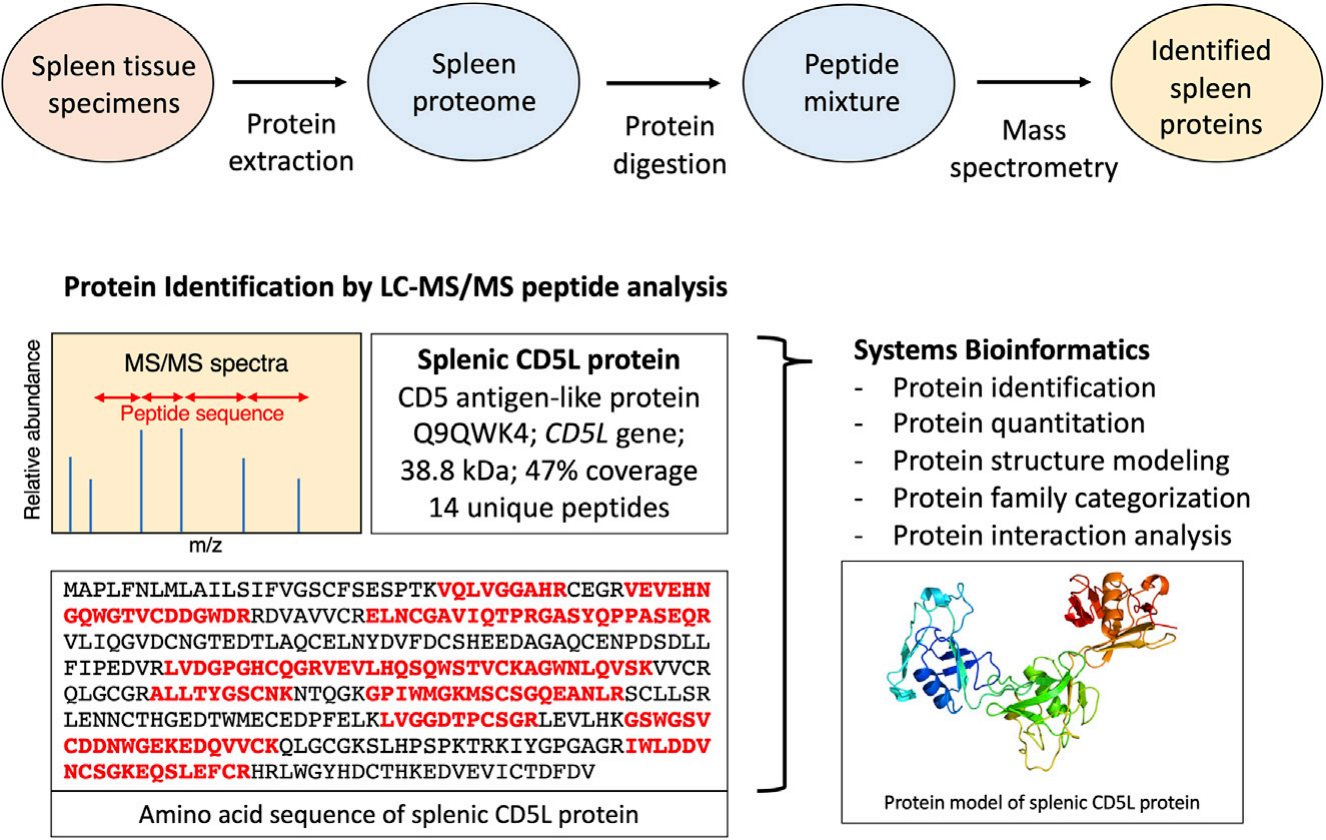
Proteomic Identification of the Splenic CD5 Antigen-like Protein in Mouse Spleen The upper panel gives an overview of the proteomic strategy used in this protocol to identify individual proteoforms in the spleen. The lower panel shows the mass spectrometric coverage of the amino acid sequence of the CD5 antigen-like protein. Unique peptides determined by LC-MS/MS analysis are marked in red and bold letters. The protein sequence was used for the molecular modeling of the splenic isoform of the CD5L protein with the help of the publicly available software program Phyre2 for protein structure prediction. The molecular model highlights the predicted positions of α helixes, β sheets, and connecting loops within the tertiary structure of the splenic CD5L protein (accession number UniProt: Q9QWK4) of apparent 38.8 kDa.

Proteomics has developed into a key biochemical methodology for the systematic cataloging of protein populations in biofluids, cells, tissues, and organs (Uzozie and Aebersold, 2018), whereby the development of highly sensitive mass spectrometric methods plays a central role in the unequivocal identification of individual proteoforms (Kang et al., 2020). This protocol describes a typical bottom-up proteomic approach (Dupree et al., 2020) that has combined tissue homogenization, protein concentration measurements, controlled protein digestion by trypsination, the removal of interfering chemicals, peptide sequencing with the help of an Orbitrap Fusion Tribrid mass spectrometer and bioinformatic analysis of proteomic data (Dowling et al., 2020). The expected outcome of a typical proteomic survey of crude tissue samples is the identification of several thousand individual proteins. For example, the recent proteomic cataloging of 12-month old mouse spleen resulted in the identification of 5,688 splenic protein species (Dowling et al., 2020). Importantly, the protocol described here for the bottom-up proteomic analysis of mouse spleen has identified a large number of splenic marker proteins. Figure 6 shows a representative finding and illustrates the proteomic results for the CD5 antigen-like protein. This protein, which was previously shown to be one of the most highly expressed splenic proteins (Uhlén et al., 2015), was identified by 14 unique peptides with a 47% coverage of the total protein sequence. The CD5L protein is mainly expressed by macrophages in lymphoid tissues and regulates mechanisms in inflammatory responses (Sanjurjo et al., 2015). Table 1 is an example of how proteomic surveys of specific tissues and organs can be employed to define the expression levels of a distinct family of proteins. The table lists the heat shock protein class of molecular chaperones (Saibil, 2013) that were identified by the described protocol for the proteomic profiling of mouse spleen. The 15 splenic heat shock proteins range in molecular mass from 11 to 96 kDa and include several sub-classes of molecular chaperones, such as small heat shock proteins (HspB, HspE), medium size heat shock proteins (HspA, HspD) and large heat shock proteins (Hsp90, HspH). These findings demonstrate the comprehensive coverage and detection sensitivity of the protocol outlined here for the proteomic profiling of spleen tissue (Dowling et al., 2020).

**Table 1:** Proteomic Identification of Heat Shock Proteins in Mouse Spleen

UniProt Accession Number	Protein Name	Gene	Coverage (%)	Peptides	Unique Peptides	Molecular Weight (kDa)
Q61699	Heat shock protein 105 kDa	Hsph1	39	26	24	96.3
P07901	Heat shock protein HSP 90-alpha	Hsp90aa1	54	43	27	84.7
P11499	Heat shock protein HSP 90-beta	Hsp90ab1	58	50	32	83.2
Q9CQN1	Heat shock protein 75 kDa, mitochondrial	Trap1	41	22	21	80.2
P17879	Heat shock 70 kDa protein 1B	Hspa1b	37	19	13	70.1
Q61316	Heat shock 70 kDa protein 4	Hspa4	56	39	36	94.1
P48722	Heat shock 70 kDa protein 4L	Hspa4l	27	17	15	94.3
P63017	Heat shock cognate 71 kDa protein	Hspa8	64	38	26	70.8
Q8K0U4	Heat shock 70 kDa protein 12A	Hspa12a	6	3	3	74.8
Q8BM72	Heat shock 70 kDa protein 13	Hspa13	3	1	1	51.7
Q99M31	Heat shock 70 kDa protein 14	Hspa14	24	8	8	54.6
P17156	Heat shock-related 70 kDa protein 2	Hspa2	26	16	4	69.6
P63038	60 kDa heat shock protein, mitochondrial	Hspd1	62	31	31	60.9
P14602	Heat shock protein beta-1	Hspb1	54	9	9	23.0
Q64433	10 kDa heat shock protein, mitochondrial	Hspe1	60	6	6	11.0

## Limitations

Although mass spectrometry-based proteomics is an excellent method for the comprehensive cataloging of large and complex mixtures of proteins from biological specimens, a given proteomic profile represents nevertheless only a snapshot of protein expression at a specific point in time. In contrast to the highly stable genome, the proteome of a specific cell is a highly dynamic entity that permanently adapts to changing physiological demands, metabolic alterations, and biochemical challenges (Walther and Mann, 2010). This limits the potential of interpreting proteomic results from a single analysis in relation to temporal changes in a biological system. Thus, studying the time course of proteome-wide alterations requires the mass spectrometric analysis of many time points, which can be both prohibitively costly and very laborious. Comparative proteomic surveys can be used to determine large-scale changes due to pathological mechanisms. This can be helpful to understand specific aspects of the molecular and cellular pathogenesis of a particular disorder, as well as for testing new therapeutic strategies and the identification of novel biomarker candidates (Dowling et al., 2016). However, as already described above, the observed changes in protein expression patterns neither give detailed information on the exact time course of pathobiochemical alterations nor provide a detailed mechanistic understanding of disease progression. Another potential limitation of the described protocol is a general issue with the usage of animal models in biomedical research. In genetic disorders, a comparable genotype might not be reflected by all pathophysiological aspects in the phenotype (Wilson et al., 2017). For example, the *mdx-type* mouse models of Duchenne muscular dystrophy, which have been utilized in this protocol, exhibit a milder pattern of fiber degeneration in most limb and trunk muscles as compared to patients suffering from dystrophinopathy (Doran et al., 2007). It is therefore important to interpret the proteomic data generated with the help of animal models with caution. Ideally, verification experiments are extended to the screening of patient biopsy material.

## Troubleshooting

### Problem 1

Difficulty with small sample size of tissues from mouse models (step 1).

### Potential Solution

For the reproducible extraction of representative protein preparations from a specific type of mouse cell, tissue, or organ, it is crucial to use large enough starting material (25 mg wet weight). If only small quantities of biological specimens can be extracted from a single individual, then samples can be pooled from several animals. Alternatively, older animals with larger organs can be utilized if the age range is suitable for the particular biomedical analysis.

### Problem 2

Interference of chemicals such as detergent in protein assays (step 7).

### Potential Solution

Various commonly used biochemicals may interfere with protein assays. In the case of detergents, which are used in this protocol during the homogenization in buffer that contains 1% (w/v) sodium dodecyl sulfate, the protein assay reagent should be supplemented with an ionic detergent compatibility reagent. For the Pierce 660 nm Protein Assay system, such a compatibility reagent is commercially available and can be conveniently added to the detection solution prior to protein measurements.

### Problem 3

Mass spectrometric profiling results in an exceedingly long list of poor proteomic hits (step 65).

### Potential Solution

During data analysis, it is crucial to define strict search parameters for the establishment of a useful list of identified protein species. Critical parameters include peptide mass tolerance, MS/MS mass tolerance and the number of missed peptide cleavages, as well as settings for certain chemical modifications such as carbamidomethylation of cysteine residues and methionine oxidation. For the unequivocal identification of individual proteoforms, another set of parameters can be concerned with the minimum number of unique peptides and the percent coverage of the total protein sequence.

### Problem 4

Low number of identified proteins with a differential expression pattern in pathological specimens (step 76).

### Potential Solution

The number of significant protein hits following the comparative proteomic analysis of normal controls versus disease samples depends heavily on the severity of the pathological phenotype. While mild pathologies might result only in a few changed proteins, severe degenerative processes can trigger alterations in the expression patterns of hundreds of proteins. Thus, if only relatively few proteins are identified that exhibit a differential expression pattern in mutant specimens from an animal model of a human disease, this can be due to a mild phenotype or an unsuitable genetic model system. In the latter case, it is advisable to try other animal models that might better mimic specific aspects of the human pathology.

### Problem 5

Poor data alignment in Progenesis QI for proteomics (step 67).

### Potential Solution

If the automatic alignment process fails to align your runs acceptably during the first round of analysis, manual alignment vectors can be included before automatic alignment is started for a second time. The combination of manual and automatic vectors is a purposeful strategy to improve alignment.

## Resource Availability

### Lead Contact

Further information and requests for resources and reagents should be directed to and will be fulfilled by the Lead Contact, Kay Ohlendieck (kay.ohlendieck@mu.ie).

## Materials Availability

This study did not generate new unique reagents.

### Data and Code Availability

This protocol has been used to generate proteomic datasets from mouse spleen. The mass spectrometric analysis of 14 separate sample runs have been deposited to the Open Science Framework repository as OSF entry “f85ve” under the following link: https://osf.io/f85ve/. This link also features a multi-consensus file of the proteomic cataloging of mouse spleen with the help of an Orbitrap Fusion Tribrid mass spectrometer.
